# Smart Nanocomposite Hydrogels as Next-Generation Therapeutic and Diagnostic Solutions

**DOI:** 10.3390/gels10110689

**Published:** 2024-10-24

**Authors:** Anna Valentino, Sorur Yazdanpanah, Raffaele Conte, Anna Calarco, Gianfranco Peluso

**Affiliations:** 1 Research Institute on Terrestrial Ecosystems (IRET)-CNR, Via Pietro Castellino 111, 80131 Naples, Italy; anna.valentino@cnr.it (A.V.); sorur.yazdanpanah@gmail.com (S.Y.); gianfranco.peluso@unicamillus.org (G.P.); 2 National Biodiversity Future Center (NBFC), 90133 Palermo, Italy; 3 Department of Experimental Medicine, University of Campania “Luigi Vanvitelli”, Via Santa Maria di Costantinopoli 16, 80138 Naples, Italy; 4 Faculty of Medicine and Surgery, Saint Camillus International University of Health Sciences, Via di Sant’Alessandro 8, 00131 Rome, Italy

**Keywords:** hydrogel, nanoparticles, drug delivery, biosensing, biomaterial

## Abstract

Stimuli-responsive nanocomposite gels combine the unique properties of hydrogels with those of nanoparticles, thus avoiding the suboptimal results of single components and creating versatile, multi-functional platforms for therapeutic and diagnostic applications. These hybrid materials are engineered to respond to various internal and external stimuli, such as temperature, pH, light, magnetic fields, and enzymatic activity, allowing precise control over drug release, tissue regeneration, and biosensing. Their responsiveness to environmental cues permits personalized medicine approaches, providing dynamic control over therapeutic interventions and real-time diagnostic capabilities. This review explores recent advances in stimuli-responsive hybrid gels’ synthesis and application, including drug delivery, tissue engineering, and diagnostics. Overall, these platforms have significant clinical potential, and future research is expected to lead to unique solutions to address unmet medical needs.

## 1. Introduction

Nanocomposite hydrogels are defined as platforms combining the properties of three-dimensional polymer networks with elements conferring higher elasticity and strength, in order to create hybrid materials that offer enhanced functionality. These platforms include a vast range of structures (e.g., membranes, sponges, fibers) among which hydrogels and nanoparticles stand out due to their remarkable design flexibility [1,2,3,4]. Hydrogels, which are highly hydrated three-dimensional networks, are exceptional scaffolds for tissue regeneration. They can closely mimic the native extracellular matrix (ECM), providing structural support and biological cues that foster tissue formation [5,6]. Hydrogels also allow the incorporation of cells, growth factors, and other bioactive molecules, making them ideal for regenerative medicine [6]. On the other hand, nanoparticles are especially well-suited as carriers for bioactive molecules, capable of efficiently encapsulating both hydrophilic and hydrophobic substances [7,8]. These versatile particles can be classified into different categories, such as organic, inorganic, and silicate nanoparticles. Organic nanoparticles, for example, can deliver drugs or genetic material [9,10,11], while inorganic nanoparticles often provide enhanced mechanical strength or unique physical properties, such as magnetic, optical, or catalytic features [12,13]. These properties can be further refined by incorporating metal ions or rare earth elements, expanding the range of possible biomedical applications [14]. In medicine, nanocomposite hydrogels are engineered to interact with biological systems with the aim of supporting, enhancing, or replacing the functions of living tissues [1,2]. By integrating nanoparticle elements into hydrogel matrices, these hybrid platforms address the limitations inherent to each material class, such as the mechanical weaknesses of hydrogels or the dispersal challenges faced by nanoparticles. This fusion results in materials with improved mechanical properties, enhanced local retention of nanoparticles, and a broader range of functionalities [15]. Furthermore, nanocomposite hydrogels can be designed with responsive features that enable them to react to a range of both internal physiological signals—such as changes in pH, redox balance, glucose levels, or enzymatic activity—and external stimuli, like temperature shifts, magnetic fields, mechanical forces, light exposure, and ultrasound waves [3,4,5,6,7,8,9,10,11,12,13,14,15,16]. By incorporating multiple stimuli-sensitive elements, nanocomposite hydrogels allow for highly personalized therapeutic strategies, such as stem cell therapy, localized drug delivery, tissue engineering and biosensing [4,5,16].

## 2. Stimuli-Responsive Nanocomposite Hydrogels and Biomedical Applications

**Figure 1:**
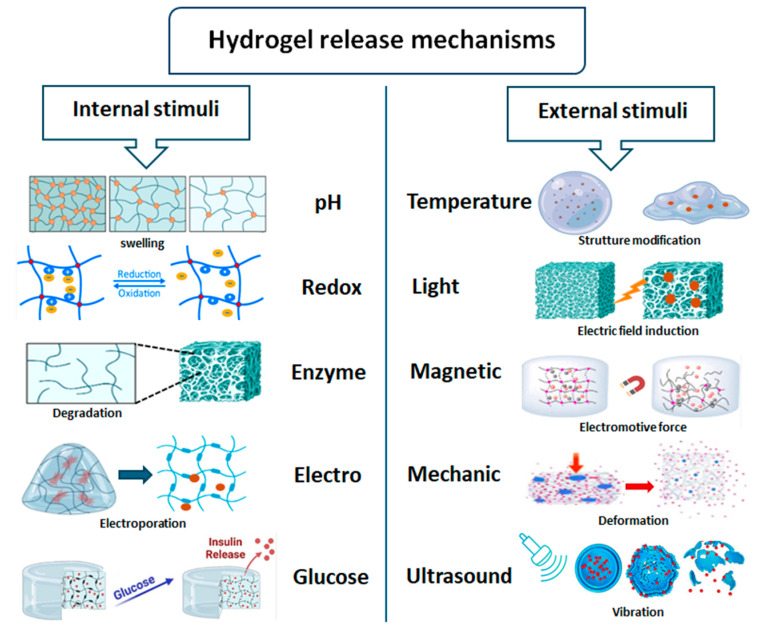
Releasing mechanism for stimuli-responsive hydrogels.

In the field of stimuli-responsive drug delivery, significant attention has been given to intrinsic stimuli that naturally occur within the human body, including pH, redox potential, enzyme activity, electric responsivity, and glucose levels [17]. These physiological signals are consistently present and, in certain pathological conditions, they can become severely dysregulated [17]. This provides a unique opportunity for intelligent delivery platforms to autonomously distinguish between healthy and diseased tissue, enabling precise and effective therapeutic interventions. By sensing these intrinsic cues, stimuli-responsive materials can act directly on the affected areas, minimizing off-target effects and improving overall treatment efficacy [17]. A key advantage of stimuli-responsive nanocomposite hydrogels is their ability to be fine-tuned for threshold sensitivity, meaning that they can be engineered to respond differently in both healthy and diseased environments. This adaptability makes them ideal for applications where precision is essential, such as cancer therapy, where the material can target abnormal tissues with minimal impact on healthy cells [17]. In contrast, nanocomposite hydrogels can also be designed to respond to extrinsic cues—external signals that are not present in tissues unless specifically applied. These include stimuli such as high temperatures, magnetic fields, ultrasound waves, mechanical forces and light irradiation [18]. This engineering strategy allows the creation of platforms that remain stable and inert in the body’s natural environment, only activating in response to an externally applied stimulus [18]. This dual approach—tuning hydrogels to respond to either internal physiological changes or externally applied stimuli—offers immense potential for developing on-demand therapeutic platforms. These systems not only ensure controlled delivery but also provide flexibility to adjust the modality of release (Figure 1), further enhancing the effectiveness of treatments.

In particular, the release mechanism of stimuli-responsive nanocomposite hydrogels involves various triggers that alter their structural properties, enabling controlled drug delivery [17]. pH-responsive hydrogels swell or shrink based on the ionization of functional groups (e.g., carboxyl or amine), adjusting to acidic or basic environments. Redox-responsive hydrogels degrade in reducing environments due to the cleavage of disulfide bonds, common in cancerous tissues. Enzyme-responsive hydrogels undergo degradation when specific enzymes cleave peptide or polymer linkages, releasing encapsulated drugs. Electro-responsive hydrogels react to electric fields by migrating charged groups, altering osmotic pressure and causing drug release. Glucose-responsive hydrogels use glucose oxidase or phenylboronic acid groups to sense glucose levels, releasing insulin in diabetic treatments. Temperature-responsive hydrogels transition at critical temperatures, swelling or collapsing to release cargo. Light-responsive hydrogels utilize photoisomerization, photodegradation, or photothermal effects to trigger structural changes or breakdown. Magnetic-responsive hydrogels contain magnetic nanoparticles that respond to external fields or generate localized heat for controlled release. Mechanic-responsive hydrogels respond to mechanical stress by altering pore structures or breaking bonds, while ultrasound-responsive hydrogels use acoustic waves to cause cavitation or heating, modulating drug release profiles. Each stimulus induces a physical or chemical transformation that enables precise, controlled drug release from the nanocomposite hydrogel [17].

### 2.1. Internal Stimuli-Responsive

**Figure 2:**
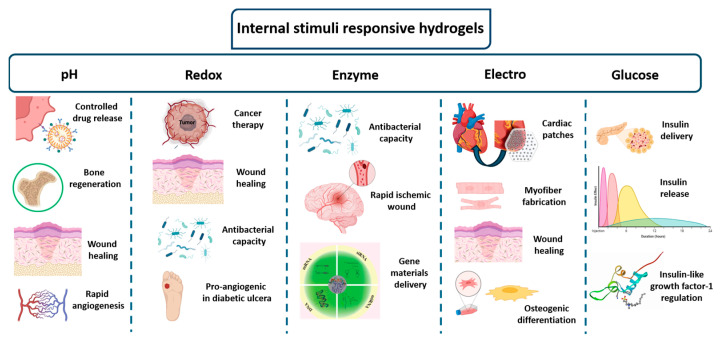
Schematic illustration of the biomedical applications of internal stimuli-responsive hydrogels.

Intrinsic stimuli that naturally occur within the human body include factors such as pH, redox potential, enzyme activity, electric responsivity and glucose levels. These physiological signals can become significantly dysregulated under certain pathological conditions [17]. Intelligent delivery platforms can differentiate between healthy and diseased tissues, allowing for targeted and precise therapeutic interventions. As shown in Figure 2, the medical applications of internal stimuli-responsive hydrogels are diverse, ranging from promoting angiogenesis to facilitating osteogenic differentiation.

#### 2.1.1. pH-Responsive

In many pathological conditions, such as tumors and inflammation, pH alterations are a common feature [19]. pH-responsive platforms leverage these changes by dynamically adjusting their structural properties in response to the acidic environments typical of these disease states. This adaptability enhances the targeted delivery of bioactive molecules to affected areas, improving the precision and efficacy of therapeutic interventions [20]. In addition to pathophysiological dysregulation, pH variations naturally occur in different anatomical sites, such as the gastrointestinal tract, where pH levels range from the acidic gastric secretions of the stomach to the more neutral or alkaline conditions of the intestines. Intracellular compartments, such as endosomes, also exhibit high acidity, facilitating the controlled release of therapeutic agents when nanoparticle species are internalized by living cells from nanocomposite hydrogels [21]. When designing pH-responsive nanocomposite hydrogels, two primary strategies are employed. The first involves incorporating pH-ionizable moieties, which alter their charge in response to the surrounding pH based on their pKa (the pH at which a molecule can donate or accept protons). The second approach involves the use of pH-cleavable dynamic covalent bonds, typically acid–labile bonds, that break in response to acidic conditions. The precise pKa of ionizable groups can be influenced by neighboring chemical groups or the conformation of surrounding components in the hydrogel’s 3D mesh [22]. Recent research has shown that the pKa of nanocomposite hydrogels made from poly(methacrylic acid) and Laponite can be adjusted based on the nano-clay content [23]. This finding holds great promise for customizing the pH-sensitivity thresholds of similar hybrid platforms, making them more adaptable for controlled and personalized drug delivery [23]. In a similar vein, scientists have engineered pH-responsive nanocomposite hydrogels by reinforcing cellulose nanocrystals with poly(acrylamido-glycolic acid), imparting pH-responsiveness through the ionization of carboxylic acid (R-COOH) functional groups in the polymer matrix [24]. These hydrogels demonstrated a controlled release of the non-steroidal anti-inflammatory drug diclofenac, with slow release in acidic conditions (pH = 1.2) and maximum release under physiological conditions (pH = 7.4). This approach indicates the potential for developing orally administrable drug delivery systems that can bypass the acidic environment of the stomach [24]. Other innovative designs incorporate pH-labile linkages to increase the design flexibility of nanocomposite hydrogels [25]. For example, recent developments have led to injectable, self-healing, and ultra-sensitive pH-responsive nanocomposite hydrogels that use Schiff base linkages between amine-functionalized silica nanoparticles and aldehyde moieties in zwitterionic polymers [26]. These hydrogels exhibit self-healing properties under physiological conditions (pH ≈ 7.4) and show substantial changes in elastic moduli, hydrolytic degradation, and drug release kinetics in response to small pH variations (≈0.2). The high sensitivity of these hydrogels makes them promising for precision drug delivery applications, particularly in cancer therapy or wound healing [26]. Beyond conventional pH-responsive designs, novel hydrogels, such as hyaluronic acid/poly-L-lysine hydrogels reinforced with mesenchymal stem cell-derived exosomes, offer multifunctional bioactive properties. These platforms have been shown to significantly accelerate wound healing, angiogenesis, and re-epithelialization of injured tissues, illustrating the therapeutic potential of integrating bioactive molecules within pH-responsive materials [24,27]. Stimuli-responsive nanocomposite hydrogels can also achieve multimodal release rates for combinatorial or sequential drug delivery. Researchers have demonstrated the spatiotemporal programming of drugs such as doxorubicin and rhodamine B by loading doxorubicin into chitosan-coated carbon nanotubes and incorporating rhodamine B into the hydrogel matrix [28]. Under physiological conditions (pH ≈ 7.4), the system exhibited distinct release rates, with higher cumulative release of rhodamine B followed by doxorubicin. This design flexibility enables the controlled release of various bioactive compounds, enhancing the therapeutic potential of each drug by tailoring the release profile to meet specific treatment needs [28]. Table 1 summarizes the pH-responsive nanocomposite hydrogels discussed, showcasing their diverse applications and design strategies.

#### 2.1.2. Redox-Responsive

The redox potential, a key biological parameter reflecting the balance of reduction-oxidation (redox) reactions, is often altered in various conditions, such as cancer, inflammation, and hypoxia [29,30]. Like pH gradients within cells, redox potential is regulated by elevated glutathione concentrations in the cytosol and endocytic organelles, creating an opportunity to trigger intracellular drug delivery. Redox-responsive nanocomposite hydrogels are designed to exploit these redox changes, commonly incorporating reduction-cleavable linkers (e.g., disulfide, diselenide, or thiol-maleimide), reactive oxygen species (ROS)-sensitive moieties (e.g., diselenide, phenylboronic ester, thioketal, or thioether), or metallic nanocomponents [31]. In a groundbreaking study, redox-responsive nanocomposite hydrogels were developed using a reversible Michael-type addition between maleimide-functionalized liposomes and arylthiol-modified 4-arm polyethylene glycol polymers [32]. This resulted in thioether succinimide adducts, which were readily degraded via a thiol-exchange reaction in the presence of glutathione, facilitating redox-responsive drug release from liposomes in thiol-rich environments. These hierarchically designed hydrogels demonstrated dual encapsulation and multimodal release of therapeutics, highlighting their potential in precision drug delivery, particularly in cancer therapy and wound healing [32]. Another innovative approach involved engineering redox-cleavable thiol-gold bonds between gold nanoparticles and thiol-containing biomaterials to create dynamic bioinks for 3D bioprinting [33]. The nanocomposite bioinks, which used gold nanoparticles as bio-responsive crosslinkers, demonstrated the ability to form stable, cell-laden filaments that adhered to constructs via inter-hydrogel crosslinks over time. This dynamic assembly–disassembly behavior, triggered by reductive, thiol-containing environments, offers exciting potential for controlled dissolution and other advanced bioprinting applications [33]. Nanocomposite hydrogels with ROS-responsive properties have also been developed for a variety of therapeutic uses, such as wound healing, pathogen prevention, and anti-inflammatory therapy. For instance, polyacrylate-coated silver nanoparticles and iron-coordinated polyglutamic acid networks exhibited ROS-responsiveness under H_2_O_2_ stimuli, enhancing antibacterial properties and improving wound healing outcomes [34]. Similarly, ceria nanocrystals, known for their ROS-responsive capabilities, were integrated into collagen-based nanocomposite hydrogels to deliver proangiogenic miRNA, showing promise for reshaping tissue phenotypes in treatments like diabetic ulcers [35]. Table 2 summarizes these redox-responsive nanocomposite hydrogel systems and their applications, demonstrating the vast potential of redox-sensitive platforms in drug delivery, tissue engineering, and wound healing.

#### 2.1.3. Enzyme-Responsive

Enzymes play an essential role throughout the human body, driving crucial biological processes that are vital for life. Their widespread presence, coupled with their tissue-specific activity and potential overexpression in certain diseases, makes them a valuable target for the design of controlled drug delivery platforms. Compared to conventional stimuli, like pH, temperature, or light, enzyme-responsive systems offer increased specificity and efficiency, making them highly suitable for precise and sophisticated drug delivery applications [36]. Enzyme-responsive nanocomposite hydrogels can be developed by using intrinsically sensitive natural polymers, such as fibrin, collagen, gelatin, and hyaluronic acid, or by modifying synthetic biomaterials with enzyme-sensitive linkers [37]. For example, hyaluronidase, an enzyme overexpressed in malignant tumors and secreted by pathogenic bacteria, has been utilized in nanocomposite hydrogels for various biomedical purposes. One approach involved hydrogels made from methacrylated hyaluronic acid and methoxy polyethylene glycol, combined with chlorhexidine diacetate-loaded lysine-based nanogels [38]. These hydrogels exhibited prolonged antibacterial activity and cytocompatibility and accelerated hemostasis and wound healing, demonstrating the therapeutic potential of enzyme-responsive systems [38]. In the context of tumor therapy, hyaluronic acid-based gels doped with iron oxide nanoparticles were crosslinked into nanocomposite hydrogels, enabling real-time monitoring of hydrogel degradation via magnetic resonance imaging (MRI) [39]. This multifunctional theragnostic platform facilitated enzyme-responsive drug delivery while allowing simultaneous tracking of the hydrogel’s degradation, highlighting its potential for personalized cancer therapy [39]. Another class of tissue-degrading enzymes, matrix metalloproteinases (MMPs), plays a key role in tissue remodeling and tumor invasion. MMPs have been targeted for the development of enzyme-degradable hydrogels, which rapidly disassemble in the presence of MMPs, releasing core crosslinked micelles that are subsequently taken up by surrounding cells. In one study, MMP-degradable growth factor-loaded nano-capsules, incorporated into MMP-degradable hyaluronic acid hydrogels, demonstrated sequential drug delivery of growth factors, leading to enhanced tissue repair in ischemic wounds [40]. In addition to growth factors, enzyme-responsive nanocomposite hydrogels have been explored for the delivery of bio-instructive nucleic acids, such as plasmid DNA (pDNA). A caged nanoparticle encapsulation process has been developed to efficiently incorporate non-aggregated pDNA nanoparticles within enzyme-degradable hydrogel networks, enabling controlled gene therapy delivery [41]. This technique holds potential for advancing treatments that rely on gene therapeutics [41]. Moreover, enzyme-responsive systems are being extended to deliver emerging gene-editing tools, like CRISPR (clustered regularly interspaced short palindromic repeats) [42,43]. These systems offer the ability to program the delivery of nanoparticles, biomolecules, and even live cells, providing a powerful approach to targeted therapies and controlled treatments for various diseases [42,43]. Table 3 summarizes the discussed enzyme-responsive delivery systems.

#### 2.1.4. Electro-Responsive

Nanocomposite hydrogels with electrical conductivity offer significant potential to enhance the electrochemical communication among cells and tissues, particularly in scenarios where electrical signal propagation is impaired due to injury. Many tissues, such as bone, cartilage, cardiac muscle, nerve tissue, and skeletal muscle, rely heavily on bioelectrical signals to function properly. By incorporating conductive nanomaterials into extracellular matrix (ECM)-mimetic networks, these hydrogels can improve both the mechanical robustness and biological activity of engineered tissues [44]. In the design of electro-responsive nanocomposite hydrogels, the construction of 3D electroactive matrices typically involves the integration of conductive inorganic nanomaterials and select conductive biomaterials, since most conventional polymers lack intrinsic electrical conductivity. Metallic nanoparticles (e.g., gold and silicon) and carbon-based nanoparticles (e.g., graphene)—whether in the form of nanosheets, nanotubes, or nanorods—are frequently used for their electrical properties. For example, gold and silicon nanowires have been incorporated into electroactive cardiac microtissues, leading to the development of biomimetic cardiomyocyte phenotypes and improved contractile machinery maturation. Additionally, 3D bioprinting techniques incorporating gold nanorods in gelatin bioinks and carbon nanotubes in gelatin-based hydrogels have enhanced cardiac cell adhesion and organization, resulting in increased cardiac phenotypic expression and synchronized contractile activity [45,46,47,48]. Conductive polymeric biomaterials, such as poly-pyrrole and polyaniline, are also widely used due to their cytocompatibility and tunable properties. For instance, electroactive nanocomposite hydrogels have been created by incorporating poly-pyrrole nanorods, which allow for electro-stimulated dexamethasone release and enhanced cell proliferation. Similarly, polyaniline-based nanocomposite hydrogels have shown success in promoting myoblast conversion into myotubes and improving wound closure in rat models [49,50,51]. Electrical stimulation has also been shown to influence stem cell differentiation, particularly toward osteogenic (bone-forming) lineages, underscoring the potential of electrical fields to guide cell behavior in tissue engineering and cell-based therapies. This highlights the critical role of 3D electroactive matrices in delivering bioelectrical cues to direct the development of specific cell types, ultimately enhancing the bio-functionality of engineered tissues [49,51]. Looking ahead, nanocomposite hydrogels with electrical conductivity are expected to play a crucial role in the development of advanced bioelectronic interfaces. These applications will depend on the continued innovation of electro-responsive hybrid platforms, which combine the benefits of mechanical strength, electrical conductivity, and biological compatibility [49,51]. Table 4 provides an overview of the electro-responsive nanocomposite hydrogel systems discussed, demonstrating their wide-ranging applications in cardiac tissue engineering, wound healing, and cell differentiation.

#### 2.1.5. Glucose-Responsive

Glucose, the primary energy source for most tissues, is intricately regulated within physiological levels in the bloodstream through a sophisticated feedback mechanism. Specialized β-cells in the human pancreas monitor and respond to fluctuations in blood glucose levels by releasing insulin in a precisely controlled manner. The delicate balance of this system can be disrupted in conditions like Diabetes mellitus, where dysregulation results in a substantial increase in glucose concentrations. Individuals with Type 1 diabetes often require regular insulin administration to compensate for the body’s inability to produce sufficient amounts [52,53,54]. Inspired by the natural processes of the pancreas, researchers have developed glucose-responsive nanocomposite hydrogels. These advanced biomaterial platforms incorporate components, such as glucose-sensitive proteins (e.g., glucose oxidase enzyme, concanavalin A) or synthetic compounds, capable of detecting and responding to glucose and its derivatives (e.g., phenylboronic acid). A common strategy involves the enzyme-dependent recognition of glucose, where glucose oxidase catalyzes glucose into outputs like pH reduction and the formation of reactive oxygen species (ROS). Pioneering research by Gu and colleagues [55] introduced self-regulated glucose-responsive hydrogels, leveraging pH-responsive matrices and hypoxia-sensitive nanovesicles in diabetic in vivo models to effectively manage both hyperglycemic and hypoglycemic episodes [55]. Moreover, the use of inorganic nanomaterials has expanded the possibilities for glucose-responsive platforms. This includes insulin-loaded mesoporous silica nanoparticles, mesoporous bioactive glass nanospheres, and metal-based nanomaterials, like MnO_2_ and ceria nanoparticles, which sense changes in glucose oxidase activity and trigger insulin release [56,57]. In addition to enzyme-dependent systems, boronic acid-based biomaterials offer a more cost-effective and durable alternative. These materials exhibit strong glucose-binding properties, bypassing the limitations of enzyme-based platforms. Recent innovations have combined maltose- and boronic acid-functionalized heparin biopolymers, resulting in 3D hybrid platforms characterized by dynamic boronate-diol interactions [58,59]. This design not only provides glucose-responsive capabilities but also enables the controlled release of insulin-like growth factor-1 (IGF-1) in response to fluctuating glucose levels [58,59]. Future developments may involve incorporating additional sensing mechanisms and utilizing synthetic biology tools to create even more sophisticated glucose-responsive systems. Recent breakthroughs, such as the creation of artificial beta cells encapsulated within nano-microvesicles [60], demonstrate the potential for self-regulating micro- and nanocarriers integrated into hydrogel-based platforms, offering improved precision and adaptability in glucose-responsive therapies. Table 5 provides a summary of the discussed glucose-responsive systems.

### 2.2. External Stimuli-Responsive

**Figure 3:**
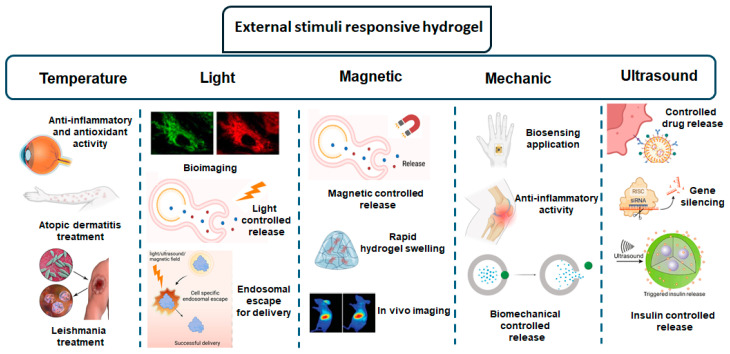
Schematic illustration of the biomedical applications of external stimuli-responsive hydrogels.

Nanocomposite hydrogels can also be engineered to respond to extrinsic cues. These extrinsic stimuli include factors such as high temperatures, magnetic fields, ultrasound waves, mechanical forces and light irradiation. When exposed to these externally applied stimulus, the hydrogels can activate, change their properties, or release therapeutic agents in a controlled manner [18]. This approach enables the creation of highly targeted and controlled delivery systems that can respond precisely to external triggers. This capability is particularly advantageous for applications that require spatial and temporal control over therapeutic interventions. Examples of these actions are summarized in Figure 3.

#### 2.2.1. Thermo-Responsive

Temperature is a widely studied stimulus in the development of stimuli-responsive platforms due to its ease of application and mild nature. Thermo-responsive polymeric materials, such as methyl cellulose derivatives, poly(N-isopropylacrylamide), and poloxamers, undergo phase transitions when exposed to temperature changes [61]. Additionally, genetic engineering can be used to create biomimetic synthetic proteins with customizable temperature responsiveness [62]. The human body’s temperature of approximately 37 °C provides an ideal environment for designing platforms that sense internal heat, leading to self-dissolving platforms or in situ hydrogel formation [63]. Thermo-responsive nanocomposite hydrogels are emerging for use in drug delivery, shape memory actuation, bio-printing, and biosensing. For instance, quantum dot nanoparticles can be incorporated as crosslinkers in these hydrogels to facilitate these applications [64]. In one example, López-Noriega and colleagues [65] developed injectable thermo-responsive chitosan hydrogels containing doxorubicin-loaded thermo-sensitive liposomes, enabling in situ thermally-triggered drug release. Similarly, Lysolipid-based thermo-sensitive liposomes (LTSLs) embedded within a chitosan-based thermo-responsive hydrogel matrix have been used for the spatiotemporal release of therapeutic agents [66]. This system allows for controlled drug scheduling and sequencing, enabling the release of multiple agents and flexible dosing with minimal invasiveness [66]. Temperature-responsive platforms have also shown promise in cryopreservation. Cold-responsive trehalose release from nanocomposite calcium alginate hydrogels has demonstrated significant improvements in islet transplantation strategies [67]. Localized drug delivery can also be achieved using thermal stimuli. For example, De Luca et al. [68] formulated eye drops containing acetylated polyethyleneimine-modified PLGA nanoparticles loaded with resveratrol (RSV-NPs) dispersed in a poloxamer 407 hydrogel. This formulation sustained the release of resveratrol for up to three days, producing antioxidant and anti-inflammatory effects in corneal epithelial cells while reversing mitochondrial dysfunction [68]. The same research group developed hyaluronic acid hydrogels containing resveratrol-loaded chitosan nanoparticles for treating atopic dermatitis. Embedding the nanoparticles in hyaluronic acid slowed the release of resveratrol and reduced oxidative damage in TNF-α/INF-γ-treated human keratinocytes (HaCaT). Additionally, pre-treatment with the hydrogel reduced the secretion and gene expression of pro-inflammatory cytokines in HaCaT cells [69]. A related study by Valentino et al. presented a localized drug delivery platform combining Hyt-loading chitosan nanoparticles (Hyt-NPs) with an in situ-forming hydrogel to provide the benefits of both hydrogels and nanoparticles. This thermo-sensitive formulation, based on Pluronic F-127 (F-127), hyaluronic acid (HA), and Hyt-NPs (Hyt@tgel), can be injected as a free-flowing solution at room temperature, forming a gel at body temperature. The system demonstrated reduced oxidative and inflammatory effects in a chondrocyte model, reduced senescence, and influenced gene expression under stress, making it suitable for osteoarthritis treatment [70]. Craig et al. explored the encapsulation of Retro-2 in an amphiphilic, thermo-responsive oligo(ethylene glycol) methacrylate-co-pentafluoro-styrene (PFG30) copolymer, which forms nanoparticle aggregates when heated above 30 °C. Encapsulation in PFG30 improved the efficacy of Retro-2 and its analogs in clearing Leishmania infections [71]. Table 6 summarizes these devices and the potential of thermo-responsive hydrogels to increase the bioavailability and efficacy of therapeutic compounds.

#### 2.2.2. Light-Responsive

**Figure 4:**
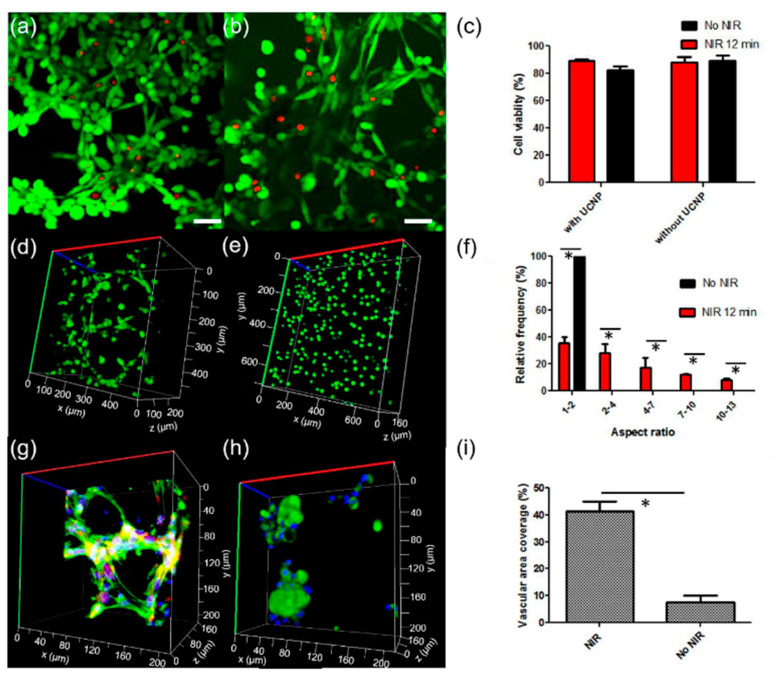
Example of hydrogel modified with light-activatable cell adhesive motifs. (**a**,**b**) Fluorescence images of live-dead staining of L929 fibroblasts encapsulated in PEG hydrogels modified with cyclo[RGDfC] modified with (**a**) and without (**b**) UCNP-PMAOs (5 mg/mL). Cells were labelled 24 h after irradiation with a 974 nm laser (10 W/cm^2^) for 12 min. Green color indicates living cells and red color dead cells. Scale bar: 50 μm. (**c**) Quantification of viability of L929 cells in (**a**,**b**). (**d**,**e**) Z-stack fluorescence images showing the morphology of L929 cultured in cyclo[RGD(PMNB)fC] modified PEG hydrogel containing UCNP-PMAOs (5 mg/mL). with (**d**) or without (**e**) NIR laser exposure. Green color indicates living cells. (**f**) Quantification of the aspect ratio (the ratio of the longest to shortest dimension) of L929 fibroblasts from (**d**,**e**). mean± s.d., n = 10 cells, * *p* < 0.05. (**g**,**h**) Z-stack fluorescence images of Human Umbilical Vein Endothelial Cells (HUVECs) within cyclo[RGD(DMNPB)fC] modified PEG hydrogels containing UCNP-PMAOs (5 mg/mL) with (**g**) and without (**h**) NIR exposure. Nucleus was stained by DAPI (blue), actin fibers with Phalloidin (green), and cell body with PECAM-1 (red). (**i**) Quantification of vascular area coverage percentage for (**g**,**h**). mean ± s.d., n ≥ 9 ROI with totals of 200–500 cells analyzed, * *p* < 0.05. Reproduced from Ref. [82] with permission from The Royal Society of Chemistry.

The use of light as an external stimulus has gained significant attention in biomedical platform design, offering precise spatiotemporal control over material behavior [72,73]. Light-responsive systems not only provide the capability for remote-controlled therapeutics delivery but also enable the sequential degradation of implantable devices in a safe, non-invasive manner. Two key approaches define these systems: photo-degradable hydrogel networks and those based on photo-reversible interactions [74,75]. Photo-degradable hydrogel networks, which often incorporate moieties like nitro-benzyl esters, enable the encapsulation and on-demand release of bioactive molecules or nanomaterials under light stimulation. In contrast, hydrogels based on photo-reversible interactions, such as azobenzene–cyclodextrin host–guest chemistry, coumarin or anthracene dimerization/cycloaddition, and engineered proteins, allow for reversible modifications in the material’s properties, such as softening or stiffening, as well as the cyclic assembly and disassembly of the hydrogel network. The flexibility of these systems is greatly enhanced when nanoparticles with antennae-like properties are integrated into nanocomposite hydrogels [76]. Within the group of light-responsive nanomaterials, photothermal and up-conversion nanoparticles have emerged as major components in hybrid platforms. Photothermal nanoparticles, including metal-based materials, carbon nanotubes, graphene oxide, and polydopamine, act as transducers, converting light into localized heat. This ability allows temperature-responsive systems to be adapted for light control, leading to applications such as remote-controlled hydrogel degradation, pulsatile therapeutic release, and thermal induction for intracellular delivery [77,78]. Metallic nanoparticles, such as gold, copper, platinum, and iron oxide, are particularly effective in photo-absorption, contributing to features like on-demand topographical changes, dynamic stiffness control, and tunable self-healing properties in light-responsive hydrogels [79,80]. Similarly, lanthanide-based up-conversion nanoparticles exhibit the unique ability to convert near-infrared (NIR) light into higher-energy UV-visible light. This property allows the combination of tissue-penetrating NIR with localized photochemical activity [72,81]. The integration of up-conversion nanoparticles (UCNPs) into nanocomposite hydrogels has led to applications involving on-demand biomolecule release, activation of cell-adhesive motifs, and complex four-dimensional control over cellular processes [82,83,84,85]. For example, Zheng et al. [82] describe a strategy for NIR controlled activation of cellular processes (3D cell spreading and angiogenesis) by embedding UCNPs in a hydrogel modified with light-activatable cell adhesive motifs. The UCNPs can convert NIR light into local UV emission and activate photochemical reactions on-demand. Such opto-regulation is spatially controllable, dose-dependent and can be performed at different timepoints of the cell culture without appreciable photodamage of the cells (Figure 4). In addition to serving as stimuli transducers, up-conversion nanoparticles can be surface engineered to reinforce the hydrogel network, making them suitable for applications in biosensing and bioimaging. The ability to organize up-conversion nanoparticles into multifunctional clusters has enabled independent activation under distinct NIR wavelengths, facilitating programmable photoactivation pathways with multiple outputs [86,87]. The described light-responsive devices and their applications are summarized in Table 7.

#### 2.2.3. Magnetic Responsive

Magnetically responsive nanocomposite hydrogels offer a promising platform for on-demand drug delivery and tissue engineering. Their unique ability to be controlled remotely using magnetic fields provides a non-invasive and precise means of modulating biomaterial properties [88]. The incorporation of metal-based nanoparticles, such as iron, cobalt, or nickel, into the hydrogel imparts magnetic properties, while biocompatibility is given by the utilization of FDA-approved materials for biomedical applications, like wüstite, magnetite, or maghemite [89,90,91]. Magnetic-responsive nanocomposite hydrogels can achieve two distinct outcomes based on their mechanism of action in response to magnetic stimuli. Firstly, static magnetic fields can induce the collective movement of nanoparticles within hydrogel-based compartments, causing mechanical deformation of the hydrogel network and triggering drug release. For example, superparamagnetic iron oxide nanoparticles (SPIONs) incorporated into Pluronic-based hydrogels loaded with anti-inflammatory indomethacin demonstrated accelerated on-demand drug release under external magnetic fields [92,93]. Secondly, magnetic nanoparticles can convert alternating current magnetic fields into heat. This magnetothermal effect has been used for wireless neurological stimulation and the creation of advanced magnetically responsive hydrogels. For example, SPION-loaded poly(N-isopropylacrylamide)-based hydrogels, subjected to alternating magnetic fields, demonstrated real-time control over swelling behavior and pulsatile drug release, showcasing the reversible network collapse/expansion in response to cyclic heating [94,95]. Although magnetically responsive hydrogels offer many benefits, some have brittle structures with poor mechanical properties. To improve their strength, especially for applications in weight-bearing tissues, researchers have been incorporating magnetic nanoparticles into double network hydrogels. For instance, functionalized SPIONs, combined with aldehyde-functionalized dextran, have resulted in injectable nanocomposite hydrogels responsive to alternating magnetic fields, demonstrating elasticity, biodegradability, and biocompatibility in vivo [96]. Finally, magnetic nanoparticles can act as magnetic resonance contrast agents, helping to create theranostic nanocomposite hydrogels. These hydrogels can both deliver drugs on demand and provide real-time imaging within the body [97]. Table 8 recaps the devices described.

#### 2.2.4. Mechano-Responsive

**Figure 5:**
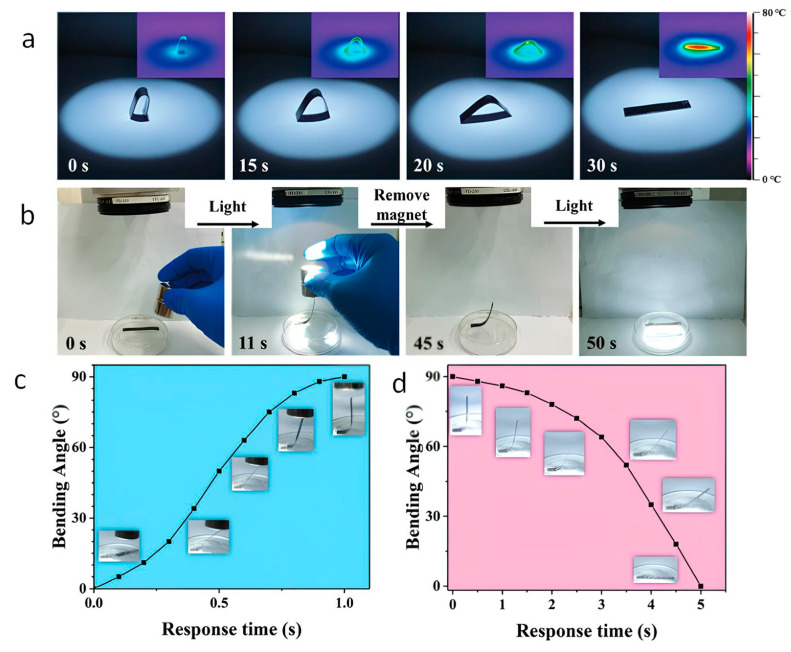
Example of magnetic to heath stimulus. (**a**) Snapshots and IR thermal images of light-response shape recovery processes; (**b**) snapshots of magnetic-and light-responsive controlled reconfiguration; (**c**) the evolution of bending behavior induced by magnetic response; (**d**) the evolution of bending behavior induced by light response. Reproduced from Ref. [112] with permission from The Royal Society of Chemistry.

Organs and tissues are constantly exposed to various mechanical forces throughout their lifespan, ranging from small-scale vibrations to large-scale pressures. At the microscale, the intricate interplay between living cells and their surrounding extracellular matrices involves individual and collective movements, creating biomechanical micro-stimulation. Cells, through these activities, probe for alterations in their microenvironment, simultaneously remodeling the 3D landscape of the extracellular matrix. This biomechanical micro-stimulation has two main outcomes: first, it activates mechano-responsive biochemical pathways in neighboring cells, altering their biomolecular outputs. Second, it triggers the release of growth factors that are transiently tethered in the extracellular matrix, perpetuating or shifting cell phenotypes and behaviors [98]. Additionally, cells are equipped with mechano-responsive protein transducers, which play a pivotal role in rapidly converting mechanical stimuli into electrochemical cues. These transducers are involved in various biological activities, including bone formation, neuron stimulation, angiogenesis, tumor tissue stiffening, and airway stretch sensing during breathing [99,100,101]. At the macroscale, mechanical forces are intrinsic to bodily functions, manifesting in heartbeats, lung expansion, facial expressions, mastication during food ingestion, and compression of joints and muscles during daily activities [102]. Moreover, tissues can undergo reprogramming in response to external mechanical stimuli, as evidenced by the application of physiotherapeutic modalities, like rehabilitation, acupuncture, and massage, which leverage kinesiology to treat acute and chronic pain or re-establish efficient body movements in physically impaired patients. Therefore, mechanical cues, in various forms such as pressure, stretching, or deformation, are highly prevalent in biological systems, influencing essential biomechanical processes [98]. Hydrogel-based platforms, designed with mechano-responsive features, have emerged as a promising avenue for mimicking the adaptable behavior of living tissues. This technology offers the potential to modulate the physicochemical properties of nanocomposite hydrogels under external mechanical cues, providing on-demand topographical adaptability, damage sensing, touch-activated drug delivery, strain-stiffening behavior, and shear-induced anisotropic alignment of hydrogel matrices [102,103]. Two primary strategies are employed to confer mechano-responsiveness to nanocomposite hydrogels. The first strategy involves the introduction of mobile crosslinks, including supramolecular and non-covalent interactions, dynamic covalent linkages, and mechanophore crosslinking agents. These mobile crosslinks enable predictable responses, such as scission, isomerization, or extrusion of small molecules, under specific force thresholds [104]. The second strategy involves the utilization of nanoparticles to provide mechano-responsive features to hydrogel networks. For example, certain nanoparticles, like poly(lactic-co-glycolic acid) (PLGA) nanoparticles, exhibit elastic deformation regimens that enhance drug release under stretching configurations. Others, such as polymeric micelles, display self-assembly equilibria that can be disrupted under mechanical forces, allowing for disassembly and subsequent reassembly of nanocarriers. Additionally, nanovesicles, like liposomes and polymersomes, can be irreversibly shredded at high strains [105,106,107]. Researchers have harnessed these mechano-responsive features to develop wearable stretch-responsive platforms. For instance, PLGA nanoparticles embedded in alginate-based hydrogel matrices showcased the potential for sustained or on-demand drug release under recurrent biomechanical movements [108]. Moreover, mechano-responsive systems designed for load-bearing tissues, such as strain-induced stiffening hydrogels, hold promise for bioactive cargo delivery in osteoarthritic patients. These hydrogels leverage supramolecular protein nanocages as sacrificial crosslinkers, with mechanical loading triggering stiffening of the network. This strain-induced stiffening behavior not only has implications for controlled drug release but also presents an avenue for providing pain relief in osteoarthritic conditions [109]. Mechano-responsive hydrogels exhibiting mechano-chromic behavior, characterized by noticeable color/fluorescence changes under strain, offer opportunities for damage reporting and strain monitoring. The incorporation of mechano-chromic agents, such as spiropyran, allows for the visual detection of structural damage and strain levels, making them suitable for theragnostic and biosensing applications [110,111]. Finally, recent advancements in mechano-responsive nanocomposite hydrogels relate the conversion of magnetic-to-heat stimuli to the engineering platform mimicking biological motors and muscles [112] (Figure 5). The described devices are schematized in Table 9.

#### 2.2.5. Ultrasound-Responsive

Ultrasound technology, once primarily used for medical imaging, is now emerging as a versatile tool for drug delivery, tissue engineering, and diagnostics. Its safety and non-invasive nature make it an asset in medical interventions [81,113]. In recent years, researchers have developed ultrasound-responsive systems that can deliver bioactive therapeutics to specific locations. Nanocarriers, like liposomes, polymeric micelles, and nano-capsules, are designed to break apart when exposed to ultrasound waves, releasing their cargo in a controlled way [114]. In particular, silica-based nanomaterials have emerged as promising candidates in ultrasound-based theranostics due to their enhanced acoustic cavitation and the ability to load sonosensitizers in mesopores [115]. In addition, alginate hydrogels are particularly useful as structural network. On these bases, researchers have developed ultrasound-responsive nanocomposite hydrogels incorporating gold nanoparticles conjugated with bone morphogenetic protein-2 that exhibit accelerated nanoparticle release under pulsatile ultrasound stimulation [116]. This innovative approach not only demonstrates the ultrasound-responsiveness of the system but also highlights the tunability of release kinetics by adjusting the surface-to-volume ratio. Growth factor-conjugated nanoparticles within these hydrogels have shown enhanced osteogenic activity, making them particularly promising for bone tissue engineering applications [117]. Ultrasound-responsive hydrogels have also been employed to achieve effective glycemic control. Insulin-loaded nano-capsules within chitosan hydrogel matrices were developed to respond to cyclic ultrasound stimuli. This strategy ensures both passive basal insulin release and on-demand release under ultrasound stimulation, leading to lowered blood glucose levels for an extended period. This approach holds great potential for long-term, non-invasive disease management [118]. Ultrasound-responsive hydrogels can also harness the locally dissipated heat generated at high ultrasound frequencies. Researchers have developed N-isopropylacrylamide (NIPAM)-based hydrogels sensitive to acoustically induced local heating, as well as copolymers with ultrasound-cleavable moieties for achieving acoustically responsive payload release [119]. Disulfide bonds, conventionally explored for redox-responsive behavior, have been demonstrated to respond to ultrasound exposure or mechanical forces, opening avenues for externally triggered delivery of siRNA cargo [120,121,122]. Calcium-loaded liposomes responding to ultrasound have been utilized to instruct calcium-dependent transglutaminase hydrogel crosslinking, presenting an innovative strategy for coupling ultrasound stimuli to enzymatic hydrogelation [123]. Additionally, certain nanoparticles, acting as ultrasound contrast agents, contribute to the development of ultrasound imageable tissue engineering constructs. For example, ZnO nanoparticles embedded in highly stretchable hydrogels enable wireless assessment of organ deformation post-implantation, providing a unique approach to monitoring shape/volume transitions in real-time [124]. Table 10 recaps the systems described.

## 3. Limitations and Challenges Associated with Nanocomposite Hydrogels

Nanocomposite hydrogels, which integrate polymer networks with nanoparticles or other nanostructures, are increasingly being explored for their potential in biomedical applications, such as drug delivery, tissue engineering, and wound healing. However, their widespread use faces several significant challenges and limitations that must be addressed before they can be fully adopted in clinical settings. One of the primary challenges is biocompatibility. While hydrogels themselves are generally biocompatible and can mimic the extracellular matrix, the inclusion of nanoparticles introduces potential risks. Some nanoparticles, such as metallic particles, like silver or gold, can generate reactive oxygen species (ROS), leading to oxidative stress, cellular damage, or inflammation [125]. This poses a risk to cell viability, particularly for long-term applications. Additionally, certain nanoparticles may be recognized by the body’s immune system as foreign invaders, leading to immune reactions or chronic inflammation. This can affect the material’s long-term safety and its ability to integrate with host tissues [126]. Another biocompatibility concern is related to the degradation of both the hydrogel matrix and the nanoparticles themselves. As the materials break down, they may release byproducts that are toxic or harmful to the surrounding tissue, further complicating their safe use in vivo [127]. Regulatory hurdles also present a significant barrier to the development and clinical application of nanocomposite hydrogels. Regulatory agencies like the U.S. Food and Drug Administration (FDA) and the European Medicines Agency (EMA), are still in the process of developing comprehensive guidelines for the evaluation and approval of nanomaterials [128]. This is particularly true for complex systems, like nanocomposite hydrogels, where both the polymer matrix and the nanoparticles must be evaluated for safety, toxicity, and long-term effects. The regulatory approval process requires extensive in vitro and in vivo testing to ensure that the materials are safe, effective, and stable under physiological conditions. Additionally, due to the novelty and complexity of these materials, there may be a lack of standardized testing methods, which can further delay regulatory approval [129]. The intricate composition of nanocomposite hydrogels—comprising polymers, nanoparticles, and potentially bioactive molecules—makes it challenging to fully characterize them, increasing the difficulty of demonstrating consistent quality and performance [129]. Large-scale manufacturing of nanocomposite hydrogels is another major challenge. While it is possible to synthesize these materials in a laboratory setting, scaling up production to meet industrial or clinical demand presents numerous difficulties. One key issue is ensuring uniformity and reproducibility during production. The precise distribution of nanoparticles within the hydrogel matrix is critical for ensuring consistent performance, yet achieving this consistently on a large scale can be challenging. The manufacturing process must also maintain the stability of nanoparticles, which can aggregate or degrade if not properly handled, leading to batch-to-batch variability. This variability can affect key properties, such as particle size, drug release profiles, and mechanical strength. Moreover, the cost of producing nanocomposite hydrogels is often high due to the specialized materials and techniques required [130]. This includes the need for sophisticated equipment and processing conditions to ensure that the nanoparticles remain stable during both production and storage. Additionally, sterilization of these materials is a crucial concern, as traditional sterilization methods (e.g., heat, UV radiation, or gamma irradiation) may alter the physical and chemical properties of the hydrogel and its embedded nanoparticles, potentially compromising their therapeutic efficacy or safety [131]. The mechanical properties of nanocomposite hydrogels also present a challenge. Hydrogels are typically known for their high water content and soft, flexible nature, making them suitable for applications in soft tissue engineering. However, many biomedical applications, such as bone regeneration or load-bearing tissues, require hydrogels with enhanced mechanical strength. Nanoparticles can improve the mechanical properties of hydrogels, but balancing this enhancement with other properties, such as elasticity and drug release kinetics, is complex. Achieving the right mechanical properties without sacrificing other essential functions is critical, as hydrogels must maintain their structure and function within the body’s dynamic environment [132]. Another significant issue is the controlled degradation of nanocomposite hydrogels. One of the advantages of hydrogels is their ability to degrade over time, which can be useful for controlled drug release or for temporary scaffolding in tissue engineering. However, with nanocomposite hydrogels, controlling the degradation rate becomes more difficult. Nanoparticles embedded within the hydrogel may interfere with the degradation process, either slowing it down or leading to incomplete breakdown of the material. This could result in prolonged retention of the hydrogel or nanoparticles in the body, potentially leading to unforeseen side effects. Designing nanocomposite hydrogels with tailored degradation rates requires careful tuning of both the polymer matrix and the nanoparticles, which adds to the complexity of their development [16]. Finally, nanotoxicity is a major concern when working with nanocomposite hydrogels. While nanotechnology offers significant benefits in improving the functionality and performance of hydrogels, the long-term effects of nanoparticles in biological systems are not yet fully understood. Certain nanoparticles, such as metallic or carbon-based nanomaterials, may accumulate in tissues or organs, raising concerns about chronic toxicity or interference with normal biological processes [133]. The body’s ability to clear nanoparticles is often limited, and there is a risk that these particles may persist in the body, leading to potential health risks over time. As research on nanotoxicity is still evolving, there is a need for further studies to fully assess the safety of nanoparticles, particularly when they are used in long-term biomedical applications.

## 4. Conclusions and Future Remarks

The field of stimuli-responsive nanocomposite hydrogels is rapidly advancing through the creation of bio-functional platforms. The integration of nanoparticles with hydrogels has unlocked remarkable design flexibility, allowing the development of biomedical devices with unique features and enhanced performance. These nanocomposite hydrogels can respond to internal stimuli—either from homeostatic states or pathological disruptions—demonstrating their potential as intelligent platforms that can recognize and address specific diseases. Their hierarchical architectures enable the controlled release of bioactive molecules at varying rates, which can be managed independently under multiple stimuli or through multi-cascade feedback systems. Additionally, external stimuli-responsive hydrogels offer reversible actuation. By integrating nanoparticles that convert stimuli into downstream signals, these hydrogels can achieve complex, cascade-type responses. Indeed, recent advancements in nanoparticles that convert magnetic-to-heat, light-to-heat, or red light-to-UV light underscore the potential of these platforms to mimic biological motors and muscles, creating sophisticated bionic systems with enhanced functionalities [112]. Despite these advancements, stimuli-responsive hydrogels and nanocarriers face significant challenges, including the lack of regulatory approval for healthcare applications. The effectiveness of these systems depends on their ability to respond to endogenous parameters, which can be affected by the complexity of biological environments and variability among patients. Developing multi-stimuli-responsive systems could mitigate these challenges by combining multiple inputs, enhancing sensitivity and adaptability across diverse patient populations [134]. Consequently, future innovations will require interdisciplinary collaboration among chemists, nanotechnologists, materials scientists, and engineers to advance these multifunctional platforms.

## Figures and Tables

**Table 1:** pH-responsive nanocomposite hydrogels.

DEVICE	APPLICATION	REFERENCE
Hydrogel Produced from PEGDA and Laponite with clay nanoparticles	Bone regeneration	[23]
Poly(acrylamido-glycolic acid) nanocomposite hydrogels reinforced with cellulose nanocrystals	pH-sensitive controlled release of diclofenac sodium	[24]
Injectable pH-responsive nanocomposite hydrogels	Precision drug delivery in cancer therapy or wound healing	[26]
pH-responsive hyaluronic acid/poly-l-lysine hydrogels reinforced with mesenchymal stem cell-derived exosomes.	Wound healing, rapid angiogenesis, and re-epithelization of injured sites	[27]
Thermo-sensitive hydrogel combined with chitosan-multiwalled carbon nanotubes using doxorubicin (DOX) and rhodamine B (RB) as model drugs.	Drug delivery system with programmed release for combined administration.	[28]

**Table 2:** Redox-responsive nanocomposite systems.

DEVICE	APPLICATION	REFERENCE
Redox-responsive nanocomposite hydrogels between maleimide-functionalized liposomes and arylthiol-modified 4-arm polyethylene glycol polymers.	Precision drug delivery in cancer therapy or wound healing	[32]
Gold nanoparticles and thiol-containing biomaterials engineered for 3D bioprinting applications.	Controlled dissolution of bio-actives	[33]
Polyacrylate-coated silver nanoparticles and iron-coordinated polyglutamic acid networks	ROS-responsiveness material with antibacterial capacity and improved wound healing	[34,35]
Ceria nanocrystals, recognized into collagen-based nanocomposite hydrogels	Delivering of proangiogenic miRNA, with action in reshaping tissue phenotype in diabetic ulcers	[35]

**Table 3:** Enzyme-responsive nanocomposite systems.

DEVICE	APPLICATION	REFERENCE
Hydrogels assembled from methacrylated derivatives of hyaluronic acid and methoxy polyethylene glycol, combined with chlorhexidine diacetate-loaded lysine-based nanogels	Antibacterial activity and promotion of accelerated hemostasis for wound healing	[38]
Hyaluronic acid-based nanogels doped with iron oxide nanoparticles	Enzyme-responsive delivery of loaded compounds and simultaneous tracking after administration	[39]
Matrix Metalloproteinases-degradable hyaluronic acid hydrogels	Sequential drug delivery of growth factors, enhancing tissue repair in ischemic wounds	[40]
Non-aggregated pDNA nanoparticles within enzyme-degradable hydrogel networks	Controlled delivery of gene therapeutics	[41]
Enzyme-responsive nanocomposite hydrogels	Controlled delivery of clustered regularly interspaced short palindromic repeats	[42,43]

**Table 4:** Electro-responsive nanocomposite systems.

DEVICE	APPLICATION	REFERENCE
Nano-wired three-dimensional cardiac patches	Cardiac patches	[46]
3D bioprinting incorporating gold nanorods in gelatin bioinks and carbon nanotubes in gelatin	Synchronize contractile frequency for cardiac applications	[47]
Hybrid hydrogels containing vertically aligned carbon nanotubes	Muscle myofiber fabrication	[48]
Nanocomposite hydrogels	Electro-stimulated delivery	[49]
Nanocomposite hydrogels incorporating poly-pyrrole nanorods	Enhancing of wound closure	[50]
Poly-pyrrole thin films formed by admi-cellar polymerization	Osteogenic differentiation of mesenchymal stem cells	[51]

**Table 5:** Glucose-responsive nanocomposite systems.

DEVICE	APPLICATION	REFERENCE
pH-responsive hydrogel matrices and hypoxia-sensitive nanovesicles	Mitigation of hypoglycemic episodes	[55]
Zeolitic imidazole framework with nanocrystals and various metal-based nanomaterials	Glucose-responsive insulin delivery	[56]
Zeolitic imidazole framework with nanocrystals and various metal-based nanomaterials	Induction of insulin release	[57]
Phenylboronic acid-based closed-loop smart drug delivery system	Therapy of diabetes	[58]
Boronic acid-functionalized heparin biopolymers resulting in 3D hybrid platforms	Controlled release of insulin-like growth factor-1	[59]
Synthetic beta cells	Insulin secretion	[60]

**Table 6:** Thermo-responsive nanocomposite systems.

DEVICE	APPLICATION	REFERENCE
Injectable thermo-responsive chitosan hydrogel containing doxorubicin-loaded thermo-sensitive liposomes	In situ thermally triggered drug release	[65]
Lysolipid-based thermo-sensitive liposomes embedded in a chitosan-based thermo-responsive hydrogel matrix	Spatiotemporal release of therapeutic agents	[66]
Nanocomposite calcium alginate hydrogels	Islet transplantation strategies	[67]
Polylactic-co-glicolyc acid- (PLGA-PEI) nanoparticles loaded with resveratrol (RSV-NPs), dispersed into poloxamer 407 hydrogel.	Release of Resveratrol for antioxidant and anti-inflammatory effects on corneal epithelial cells.	[68]
Hyaluronic acid hydrogels containing resveratrol-loaded chitosan nanoparticles	Treatment of atopic dermatitis.	[69]
Thermo-responsive Pluronic-Hyaluronic hydrogel containing hydroxy-tyrosol-chitosan nanoparticles	Localized drug delivery platform	[70]
2-(((5-Methyl-2-thienyl)methylene)amino)-N-phenyl-benzamide (also called Retro-2) in oligo(ethylene glycol) methacrylate-co-pentafluoro-styrene (PFG30) copolymer that forms nanoparticle	Treatment of Leishmania	[71]

**Table 7:** Light-responsive nanocomposite systems.

DEVICE	APPLICATION	REFERENCE
Nanoparticles with antennae-like capabilities into hydrogel formulations	On-demand release under light stimulation.	[76]
Carbon-based nanotubes and graphene oxide with efficient photothermal conversion of near-infrared (NIR) light	Remote-controlled hydrogel degradation, pulsatile payload release, and thermal induction of endosomal disruption for intracellular delivery	[77,78]
Lanthanide-based up-conversion nanoparticles into gel matrix	Photochemically-active blue photonic irradiation for triggered release	[81]
Up-conversion nanoparticles serving as stimuli transducers surface-engineered for network reinforcing in nanocomposite hydrogels	Biosensing and bioimaging applications	[86]

**Table 8:** Magnetic-responsive nanocomposite systems.

DEVICE	APPLICATION	REFERENCE
Superparamagnetic iron oxide nanoparticles (SPIONs) incorporated into Pluronic-based hydrogels loaded with indomethacin	Accelerated on-demand drug release under external magnetic fields	[93]
SPION-loaded poly(N-isopropylacrylamide)-based hydrogel subjected to alternating magnetic fields	Real-time control over swelling behavior and pulsatile drug release	[96]
Theranostic nanocomposite hydrogels with magnetic nanoparticles	On-demand drug delivery capacity and in vivo imaging features in a single administrable platform.	[97]

**Table 9:** Mechano-responsive nanocomposite systems.

DEVICE	APPLICATION	REFERENCE
PLGA nanoparticles embedded in alginate-based hydrogel matrices	Sustained or on-demand dexamethasone release under recurrent biomechanical movements	[108]
Strain-induced stiffening hydrogels with protein nanocages as sacrificial crosslinkers	Cargo delivery of anti-inflammatory drugs in osteoarthritic patients.	[109]
Mechano-responsive hydrogels exhibiting color/fluorescence changes under strain	Visual detection of structural damage with theragnostic and biosensing applications	[111]
Magnetic to light bionic system	Replication of mechanical movements	[112]

**Table 10:** Ultrasound-responsive nanocomposite systems.

DEVICE	APPLICATION	REFERENCE
Nanocomposite hydrogel with mesoporous Silica Nanoparticles	Controlled-Release Drug Delivery	[115]
Alginate hydrogel incorporating gold nanoparticles conjugated with bone morphogenetic protein-2	Accelerated nanoparticle release under pulsatile ultrasound stimulation	[116]
Ultrasonically burstable capsules	Sequential release of nanoparticle payloads	[117]
Insulin-loaded nano-capsules within chitosan hydrogel matrices	On-demand insulin release under ultrasound stimulation, leading to lowered blood glucose levels for an extended period.	[118]
Ultrasound-responsive NIPAM-based hydrogels	Controlled release of large molecules	[119]
Carbon nanotubes in ultrasound-responsive matrix	Delivery of siRNA and Potent Gene Silencing	[122]
Calcium-loaded liposomes into a calcium-dependent transglutaminase hydrogel crosslinking	Innovative strategy for coupling ultrasound stimuli to enzymatic hydrogelation	[123]
ZnO nanoparticles embedded in highly stretchable hydrogels	Wireless assessment of organ deformation post-implantation,	[124]

## Data Availability

No new data were created or analyzed in this study.
